# Exploring synthesis and applications of green nanoparticles and the role of nanotechnology in wastewater treatment

**DOI:** 10.1016/j.btre.2024.e00830

**Published:** 2024-01-26

**Authors:** Shreya Rathod, Subham Preetam, Chetan Pandey, Sweta Parimita Bera

**Affiliations:** a School of Sciences, P P Savani University, Surat, Gujarat, 391425, India; b Institute of Advanced Materials, IAAM, Gammalkilsvägen 18, Ulrika, 59053, Sweden; c Daegu Gyeongbuk Institute of Science and Technology (DGIST) Daegu, 42988, Republic of Korea; d Department of Botany, Hindu College, University of Delhi, New Delhi, 110007, India

**Keywords:** Green nanotechnology, Nanoparticles, Green NPs, Wastewater, Bioremediation

## Abstract

Current research endeavours are progressively focussing towards discovering sustainable methods for synthesising eco-friendly materials. In this environment, nanotechnology has emerged as a key frontier, especially in bioremediation and biotechnology. A few areas of nanotechnology including membrane technology, sophisticated oxidation processes, and biosensors. It is possible to create nanoparticles (NPs) via physical, chemical, or biological pathways in a variety of sizes and forms. These days, the investigation of plants as substitutes for NP synthesis methods has drawn a lot of interest. Toxic water contaminants such as methyl blue have been shown to be removed upto 70% by nanoparticles. In our article, we aimed at focussing the environmental sustainability and cost-effectiveness towards the green synthesis of nanoparticles. Furthermore it offers a comprehensive thorough summary of green NP synthesis methods which can be distinguished by their ease of use, financial sustainability, and environmentally favourable utilization of plant extracts. This study highlights how green synthesis methods have the potential to transform manufacturing of NPs while adhering to environmental stewardship principles and resource efficiency.

## Highlights

• Nanotechnology has opened up new possibilities in bioremediation and biotechnology fields, like extends to membrane technology, advanced oxidation processes, and biosensors.
• Nanoparticles (NPs) can be generated in various shapes and sizes through physical, chemical, or biological means. Conventional methods using potent reducing agents lead to energy inefficiency, reduced yields, high costs, and environmental harm.
• Microorganisms (bacteria, fungi, yeast, algae) and plants offer alternative pathways for NP synthesis.
• Sustainable nanoparticle synthesis methods are preferred to align with environmentally friendly principles.
• This review provides an overview of environmentally friendly NP synthesis using plant extracts, focusing on simplicity, cost-effectiveness, and environmentally beneficial applications.

## 1 Introduction

Nearly two thirds of the earth's surface is covered with water, which is essential to all life forms. Water molecules, when interact with organic, inorganic substances, pathogenic microorganisms, heavy metals and contaminants, perform various physical, chemical, or biological functions [1]. Contamination of waterbodies makes them a harmful source for water consumption. The main factor which demands an urgent need for sustainable management of water resources is pollution. [2]. Scarcity of clean drinking water and it's easy availaibility is one of the biggest challenges of the 21st century. The globalization of raw ingredient and petrochemical companies has caused emission of hazardous inorganic and organic contaminants in various water bodies [3]. More polluted than clean sources of water are affecting society's standards of life [4]. Wastewater surveillence has been deemed crucial in identifying the epidemiology of viruses such as SARS-CoV-2 [5]. Although the virus did not cause infection through this source, the excrement that was expelled into the waterbodies presents a concerning risk for the introduction of additional pathogens that can cause potential waterborne infections. Drinking unclean water is the primary cause of ninety percent of diseases in impoverished nations. The polluted wastewater may be separated into two categories: (i) municipal wastewater, which comes from household activities, and (ii) non-residential wastewater, which is generated from sewage and industrial effluents. According to the 2015 UN-Water report, there are 1% of suspended colloidal dissolved particles in wastewater [6]. Residential wastewater comes from public housing which comprises of 99.90% water, 10% particles and nutrient-rich organic materials that decompose and are broken down by microbes. Furthermore, the main sources of non-residential or urban wastewater are commercial, industrial and agricultural activities, and these sources also influence the effluent's composition [7]. To provide cleaner and non-hazardous water resources, industrial emmisions of dangerous dyes like methylene blue must be stopped [8].

An urgent need for sustainable wastewater management is desperately needed to ensure that everyone has access to a clean and safe water supply. A wide variety of traditional and nonconventional treatment methods are available for the purpose of removing pollutants from different types of wastewaters [9]. These methods have two disadvantages: they are not always successful and demand a large initial financial investment. This challenge also comes with the requirement of specialized operating conditions, high maintenance costs, and high energy requirements [10]. However, nanotechnology significantly promises the development of next-generation wastewater technologies and it has the potential to displace current wastewater treatment technologies [11]. The world's research community is interested in conducting exploratory experiments to enhance the characteristics of nanotechnology because of the swift advancements in science and technology [12]. Nanotechnology is the science of manipulating matter so that any of its dimensions can be contained within the nanoscale range or 1–100 nm. Nanoscale particles have extensive surface area, high surface energy, and quantum confinement and also exhibit a wide range of unique optical, magnetic, and electrical features. Recent advances in wastewater management have greatly benefited from special properties of nanomaterials such as enhanced catalysis, adsorption capacities, and high reactivity [13]. Industrial wastewaters have been widely treated by photocatalysis with the use of nanoparticles, in particular titanium dioxide that is produced chemically. Nanoparticles provide a better delivery system than nanotechnology on the whole. Titanium dioxide is used for protection from UV light for a longer duration and comes under efficient nanomaterials. When very toxic, expensive and hazardous substances are required for the chemical production of NPs, their environmental release shows serious ecotoxicological issues [14]. To create nanoparticles from biodegradable substances, use of plant extracts, microorganisms, and enzymes provides an upfront ecologically acceptable method [15]. The best method for creating nanoparticles is to use plant extracts because they reduce the likelihood of related contamination and speed up the reaction process. They also help preserve the cell structure [16]. Green synthetic nanoparticles have recently been used to clean wastewater by a relatively small number of researchers [17]. Numerous publications highlight the potency of nanoparticles in wastewater treatment. Our main focus has been on non-hazardous green chemistry approaches where we tried to keep up with current information that summarises the effectiveness of currently practised novel techniques and their findings.

## 2 Role of nanotechnology in wastewater treatment

The science and technology of various nanostructures have grown to be an important field of study. Human civilization has advanced as a result of it, and it will continue having a substantial commercial influence in the future [18]. Norio Taniguchi first coined the term nanotechnology in 1974 [19]. He asserted that the application of nanotechnology allows materials to be divided, consolidated, and deformed by a single atom or molecule. Nanomaterials have high reactivity, functionalization, a large specific surface area, size-dependent properties, and other characteristics that make them appropriate for wastewater treatment [20]. Improved sea and brackish water desalination, safe wastewater reuse, water disinfection, and decontamination are the main goals of nanotechnology-based water treatment technologies [21]. This includes the removal of pollutants through biosorption and nanoadsorption, the detection of contamination through nanosensors, and various membrane technologies such as reverse osmosis, nanofiltration, ultrafiltration, and electro-dialysis, to name a few [22]. Nanoparticles exhibit distinct optical, electrical, and magnetic properties compared to the bulk one due to its huge surface area and high surface energy.

Due to the remarkable and significantly altered properties at the nanoscale, materials have a wide range of potential applications in wastewater treatment [23]. The properties and functionalities of materials can change as they transition from being bulky to becoming nanostructured [24]. If two parameters-the length scale of the material and the structural arrangement of atoms or molecules-are moulded appropriatey, the qualities of materials. The table below shows different technologies that have been utilised for treating wastewater and various methods used to synthesize nanoparticles have been discussed in detail as shown in Table 1.

**Table 1:** Different technologies utilized for treating wastewater.

Primary	Secondary	Tertiary	Nanobased
Coagulating	Aerobic	Evaporation	Photocatalysis
Flocculation	Anaerobic	Oxidation	Nanofiltration
Centrifugation		Ion Exchange	Nanocomposites
Screening		Distillation	Nanoadsorbents
Centrifugal separation		Reverse Osmosis	Clay and Zeolites
		Adsorption	

### 2.1 Photocatalysis

The rate of a chemical reaction can change under the influence of ultraviolet, visible, or infrared radiation [25]. A material that absorbs light and aids in the chemical conversion of the current reaction partners is called a photocatalyst. It is a surface phenomenon that often involves the five steps listed below: (i) reactions that spread across it; (ii) reaction products that stick to it; (iii) surface transformation; (iv) a procedure that removes the products from the catalyst's surfacel; and (v) product evaporation [26].

The greatest photocatalysts are nanostructured semiconductor materials because of the large amount of photogenerated electrons and holes accessible at the surface. Inorganic and organic pollutants like microorganisms and heavy metal ions can both be removed using photocatalysis [27]. Obtainence of chemical energy from photon energy has been used in heterogenous photocatalysis which is an advanced oxidation process [28]. Studies on the semiconductor TiO_2_ for heterogenous photocatalysis of wastewater have also been done efficiently [29]. Photocatalysis is a viable method that can be used as a promising source for utilizing light to initiate spontaneous and non-spontaneous reactions [30]. Methylene blue degradation potential of copper nanoparticles reveals their photocatalytic activity. Additionally, this substance has strong anti-fungal properties [31].

### 2.2 Nanofiltration

An other recommended technique for purifying sewage is nanofiltration. There may be other applications for membranes in this particular type of membrane technique [32]. The process of membrane filtration is pressure-driven. It allows relatively pure water to flow and selectively blocks the entry of harmful substances like organics, nutrients, turbidity, microorganisms, inorganic metal ions, and other substances that deplete oxygen [33]. Strict laws and regulations pertaining to water quality are the consequence of technological breakthroughs [34]. The use of microfiltration and ultrafiltration for wastewater treatment has been documented in several studies. Various microporous membranes have been used and are created by solution casting in microfiltration and ultrafiltration. These membranes remove heavy metals are resourceful for the treatment of wastewater. Carbon derivatives are one of the most used adsorbents. These adsorbing membranes are considered essential due to the presence of the functional adsorption groups on the membrane material which remove heavy metals [35].

Membrane processes may be broadly categorised into four basic classifications: (i) microfiltration, (ii) ultrafiltration, (iii) nanofiltration, and (iv) reverse osmosis membrane filtration. The finest solution for water filtration is nanofiltration membranes [36]. Polyamide membranes, which have a lower salt rejection and a range of cut-offs depending on the molecular weight of a species. These membranes have a rejection rate of over 92%. The relatively new technology utilised in wastewater treatment systems is known as the nanofiltration membrane [37]. The removal of pollutants from wastewater and pulp-bleaching effluents, textile effluents, demineralization in the dairy industry, and the separation of medicines from fermentation broths have all been made possible by nanofiltration technology in recent years.

### 2.3 Nanosorbents

Nanosorbents have been widely employed to remove organic colours, heavy metal contaminants from water and wastewater [38]. Because of their small size and high surface area-to-volume ratio, nanoparticles are special. They are particularly suitable for certain types of contaminants as they are biocompatible and easily adaptable [39]. Due to the production of metal-ligand precipitation, metal oxide at the nanoscale exhibits a higher level of adsorption than at bulk size. The wastewater's pH level impacts how effectively metal pollutants are removed via an adsorption method. With an increase in pH, wastewater treatment became more effective at removing heavy metal ions. Increasing the pH of the wastewater and the attractive relationships between highly charged metallic ions can improve decay at the nanosorbent surface [40]. Furthermore, when the number of negatively charged sites on adsorbents rises, so do the number of the negative sites. Based on their use or surface properties in absorption applications, nanomaterials may be categorised into many groups, such as (i) carbon nanomaterials, (ii) silicon nanomaterials, (iii) nanomaterials, and (iv) nanoparticles as adsorbents [41].

## 3 Methods for synthesizing nanoparticles

Adaptation of several practical methods to produce nanoparticles by varying their shapes, sizes, and chemical composition has been an attractive area of research [42]. The results of various multidimensional 1-D, 2-D, and 3-D meso‑structures is also caused by the interaction of nanoparticles with organic molecules, which stimulates shared behavior and sparks inquiries. The two main ways of synthesizing nanoparticles are top-down and bottom-up approaches [43].

The physical processes that break down bulk particles into minute ones, resulting in nanoparticle formation, are included in the top-down approach. On the other hand, the bottom-up approach uses biological and chemical processes [44]. Some examples include using sol-gel, laser pyrolysis, plasma spraying, aerosol-based techniques, and green synthesis methods [45], as shown in Table 2. They have little or no adverse effect on the surface structure. The nanostructures are generated by adding one atom to another, and the production of nanoparticles begins at the fundamental atomic or molecular levels. To create nanostructured materials with homogeneous structures and distributions, the bottom-up method is better than the top-down approach [44]. Cu_2_O nanoparticles synthesized and impregnated in textiles show eligible anti-microbial properties which can also be an asset to be used in the treatment of contaminated water supplies [46] Synthesized Selenium Nanoparticles (SeNPs) also exhibit remarkable anti-fungal activity [47].

**Table 2:** Nanomaterial synthesis methods and approaches.

Biological Methods	Physical Methods		Chemical Methods
Green Synthesis	mechanical technique	vapor technique sputtering	Sol-Gel
Utilizing plant parts like seeds, stalk, stem, leaf, and latex	mechanical milling	vapor deposition in physical form	Chemical Vapourisation
utilizing microbes like bacteria, yeast, and fungus	vapor deposition in physical form	Sputtering	Covalent approach
		Laser ablating	
		Laser devolatilization	Spray pyrolysis

### 3.1 Mechanical milling technique

**Fig. 1:**
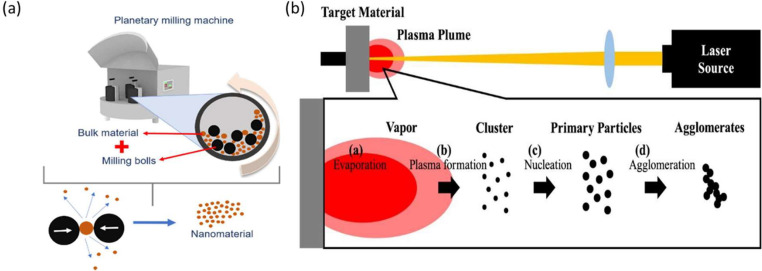
(a) Ball Milling method for Nanoparticle synthesis, (b) The process of Laser ablation [49].

Powders are the main product of this process. The surfaces of tiny particles extracted from bulk materials are what make up fine particles. When an agitator or high-speed jets are introduced, they rub against one another under extremely high pressure and friction, forming them [48]. Due to the route's extremely high energy usage, the parent bulk material is continuously exposed to structural flaws, chemical imbalances, and elastic strain. Hence, as a result, nanostructures are created. Ball milling is an excellent example of mechanical milling [49]. Nanomaterials are made using this automated technique. During this process, the components are ground in a closed container [50]. When grinding, tiny glass, ceramic, and stainless steel pebbles create sheer stress. The mechanical milling synthesis of nanoparticles by the ball milling method is demonstrated in Fig. 1(a).

### 3.2 Vapor deposition in physical form

Nanostructures in the gas phase are produced via physical vapor deposition, that involves heating the original bulk material with a focused electron beam [51]. Atoms are removed from the target material in the first step by utilizing a high-energy ion source in the presence of a vacuum and an inert gas, commonly argon. A high-energy source is applied to the target material, causing the target's surface to evaporate [52]. After that, the vaporised atoms move in the direction of the substrate's surface and settle within the chamber. The reaction does not occur without the deposition of a metal oxide, carbide, or nitrite. A thin deposition layer is formed by the vaporised atoms as they get to the substrate's surface [53]. When creating the final product from the parent entity, such as through sputtering, laser ablation, laser pyrolysis, etc., this approach does not rely on any catalytic contact during the synthesis process. Additionally, no chemical reaction occurs from the beginning to the end of the process [54].

### 3.3 Sputtering

**Fig. 2:**
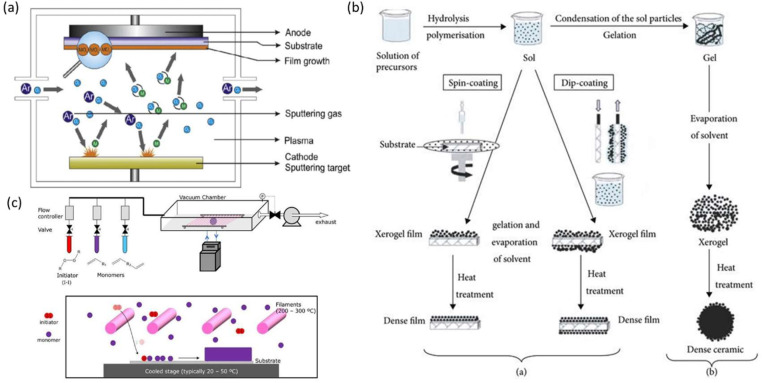
(a) The process of Sputtering representing Ar with motion directions (b) Schematic diagram showing the Sol-Gel method, (c) Fig. 5: Process of nanoparticle synthesis by chemical vaporization [66].

The process of sputtering uses the energy of plasma, or partly ionised gas, on a target's (cathode) surface. One by one, it extracts and places the material's atoms on the substrate [55]. To do this, plasma is produced by ionizing pure gas (typically Argon) using a potential difference (pulsed DC) or electromagnetic excitation (MF, RF). Ar+ ions are the building blocks of plasma, and a magnetic field confines and accelerates them around the target. When an ionised atom hits a target, it transmits its energy and tears an atom, and the ton of atoms have enough energy to project onto the substrate [56]. This method does not depend on any catalytic interaction during the synthesis method for producing the final product from the primary organizations, such as by laser ablation or pyrolysis [57]. The schematic diagram of sputtering method is shown in Fig. 2(a).

### 3.4 Laser ablation

Lasers have been used and studied extensively for a wide range of purposes, including laser ablation, ever since they were discovered [58]. The application of laser ablation to generate nanomaterials for gas sensing did not begin until the mid-1990s, despite the fact that the first experimental study on the subject was published in 1963 [59]. A laser ablation heating method melts and evaporates materials like metals and compounds while creating nanostructures in a high vacuum environment [60]. Laser ablation is the removal of materials from a surface by laser irradiation. In contrast to "laser evaporation," which is the heating and evaporation of materials in a state of thermodynamic equilibrium, "laser ablation" emphasizes the nonequilibrium vapor/plasma conditions induced on the surface by a powerful laser pulse [61]. The figure demonstrating laser ablation technique is shown in Fig. 1(b).

### 3.5 Laser devolatilization

In the process of laser devolatilization, the vapourised reactant material is broken down with the help of an intense laser beam in an inert gas environment [62]. This process involves colliding reactant and inert gas atoms, forming a nanostructured film, which is then deposited on the substrate [63]. In this technique, species that have been laser-vaporised proliferate and form nanoparticles in the form of a background gas [64].

### 3.6 Sol-gel

Sol-gel is a chemical process that uses gelation, precipitation, and then calcinations to create nanoparticles. The gel's constituent parts are stabilized sols, which are frequently seen as colloidal aggregates of tiny metal oxy‑hydroxy particles in an aqueous solvent. However, this aggregation is easily dispersed if any capping agents are present [65]. Regulated condensation and hydrolysis play a critical role in the gelation process of the system. Different physical and chemical factors like temperature, pH, and the concentration of metal ions in the precursor solution affect the dissolving duration [66]. Since the porosity of gel changes with pH, it significantly impacts the surface characteristics of the produced material. As it is customary for creating nanostructures, metal alkoxides are used as precursors in an organic solvent [67]. The chelate, which can stabilize metal cations, is occasionally used in place of alkoxides as a precursor. Chelation makes multicomponent gels easier to prepare, such as a combined titanium dioxide and silicon dioxide gel [68]. The schematic representation of the sol-gel method is shown in Fig. 2(b).

### 3.7 Vaporization of chemicals

A very pure, well-performing nanostructural thin film is created using the chemical vapor deposition procedure. In this method, heating is used to deposit the precursor onto a substrate's surface. This is followed by evaporation to produce vapor, and the deposition occurs through a chemical reaction in a vacuum to create a difference in chemical characteristics between the precursor material and the result [69]. A template is initially generated on the substrate's surface, followed by the formation of nanostructures on the template. The reaction temperature, reaction rate, and precursor concentration during the deposition event are all constant factors that affect the creation of the nanostructure. Despite some limitations from the process's more significant temperature requirement, this approach generally makes it easier to cover nanostructures uniformly on the surface of the substrate [70]. The working process of vaporization of chemicals for synthesising nanoparticles is explained in Fig. 2(c) .

### 3.8 Covalent approaches

This colloidal technique is a type of chemical precipitation in which several ionic solutions are made to blend together and then precipitated under regulated temperature and pressure conditions, as well as by adjusting the concentration of the reagent and capping agents. This technique needs a stabilizer to prevent agglomeration, primarily caused by Vander Waals forces between colloidal nanoparticles [71]. Stabilizers can be electrically or sterically stabilized by Coulombic repulsions by surfactant adsorption onto the surfaces of the nanoparticles, respectively. The creation of metal and metal oxide nanoparticles using this technique is quite common and beneficial in various chemical and pharmacological domains.

### 3.9 Spray pyrolysis

Spray pyrolysis is a standard chemical deposition method to create thin films containing nanomaterials. Many factors like spray rate, the substrate's temperature, and the concentration of the chemical solution, influence the creation of thin films [72]. By managing the size of the droplets and how they are distributed throughout the substrate while spraying, the effectiveness of deposition can be increased. Using the facilities for doping various elements in spraying solution in arbitrary proportions, nanostructures can be formed on the substrate as a film in a straightforward manner [73]. Compared to other vapor deposition processes, this approach has some advantages that make it a more suitable method for industrialization because the quality of the substrate, it's dimension, and surface qualities are not constrained.

## 4 Green nanotechnology as a better alternative to traditional methods

**Fig. 3:**
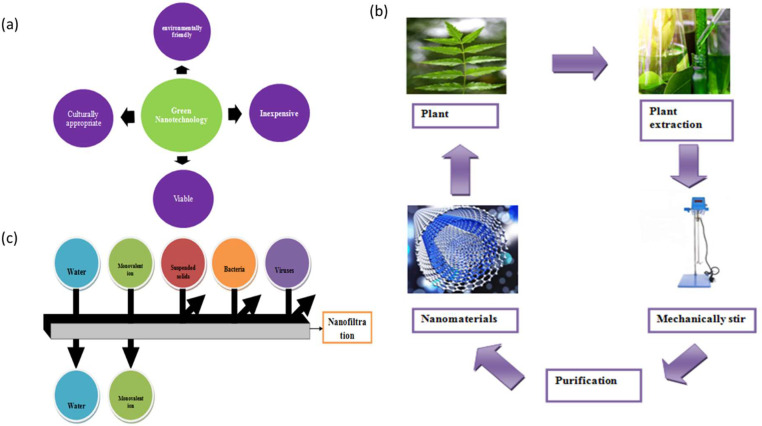
(a) Green nanotechnology and its benefits, (b) Green Synthesis of nanomaterials by plant scores, (c) Schematic diagram showing the process of Nanofiltration [71,74,79].

Green nanotechnology employs several biotechnological techniques to produce nanomaterials (or nanoparticles) utilizing biological means, such as bacteria, fungi, or plants [74]. The resultant nanoparticles are safe for the environment and nontoxic. Safer, more environmentally friendly nanomaterials may be produced and processed more easily by using the concepts of green chemistry in nanoscience. Green chemistry is used in green nanotechnology to design and improve small- and large-scale procedures for producing nanomaterials and to employ nanomaterials across a range of sectors [75]. Additionally, it intends to educate people about the characteristics of nanomaterials concerning toxicological concerns and the development of multifunctional nanomaterials that can be employed in high-capacity goods that could be hazardous to human health and the environment as shown in Fig. 3(a) [76]. Particularly, it aims to create synthesis methods and systems that can replace the requirement for dangerous chemicals while improving the effectiveness of these current synthesis techniques. It also offers guidelines for assessing ecological risks and hazards in relation to design to ensure that the nanoproducts as they have been synthesized are safer [77]. Plant derived silver nanoparticles are useful in controlling *Aedes* mosquito populations whose larva is found in stagnant wastewater [78] Green synthesized Iraqui Date Platinum nanoparticles (PtNPs) show potent anti-microbial properties against Pseudomonas and Streptococcus species of bacteria [79].

## 5 The green synthesis of nanomaterials

Green synthesis has the potential to significantly alter industrial-scale nanomanufacturing processes by creating novel nanomaterials using green chemistry principles [80]. In the upcoming years, greener nanomaterials may become increasingly important in nanomedicine as creative drug carriers, among other applications. Green synthesis does not require expensive machinery, and the synthesis process is comparatively faster as it works at ambient temperature and can be scaled easily [81]. Utilizing nontoxic, biodegradable, economical resources, and energy-efficient processes are the fundamental tenets of green chemistry. While the synthesis of nanomaterials solely from organic materials has garnered significant attention in the field of nanomanufacturing, other chemical methods are also included in the category of green chemistry-based synthesis strategies. Although using only organic items to create nanomaterials has drawn a lot of interest in nanomanufacturing, other chemical processes also fall within the green chemistry-based synthesis techniques. Scientific publications have shown that green synthesis methods can provide nanomaterials that are just as effective and chemically equivalent to those made otherwise. Heat, pressure, severe presuure, and electricity must all be transformed in order to perform chemical synthesis. However, microbe-mediated syntheses are not feasible on a larger scale since they need maintenance in the lab. In a manner comparable to chemical synthesis, it is also feasible to modify the properties of nanomaterials by manipulating the reaction conditions, such as temperature and pH concentration. Nanosensing, nanomedicine, nanotherapeutics, energy storage, and other fields are most commonly used for these more environmentally friendly nanomaterials shown in Fig. 3(b).

Using a variety of methods, several multifunctional green nanomaterials have been produced, and their potential applications have been studied. These materials have specific compositions, sizes, morphologies, and crystallinities. Alkaloids, flavonoids, phenolic acids, and terpenoids are some of the primary and secondary metabolites found in the plant's extract. These substances oversee reducing or converting bulk materials into nanoparticles. These metabolites play a key role in redox reactions that produces environmentally friendly, green nanomaterials. An analysis of the literature revealed that the synthesis phase, where toxic waste is more likely to be produced, is the key factor in the influence of nanomaterials on the life cycle. Numerous academics have mentioned a lack of transparency, significant variations in data collection, and various approaches. The nanomanufacturing processes linked to detrimental human and environmental repercussions should be thoroughly examined when assessing nanomaterials for their greenness for sustainable development.

The methods used for cleaning wastewater are expensive, chemically intensive, and, to some extent, environmentally hazardous [82]. Traditional treatment systems need a large amount of capital and maintenance costs since they just transport pollutants from one phase to another, not completely degrading them into an environmentally benign end [83]. Significant drawbacks of these methods include sludge formation, handling, high cost, membrane deformation, and problems with disposable materials. However, non-biodegradable contaminants pose a significant concern because they typically require immediate attention.

Environmentally friendly wastewater treatment techniques may be created using nanotechnology-based membranes, adsorbents, and catalysts [84]. The application of nanotechnology to the treatment of industrial wastewater can thus be summarised as follows: using metal and metal oxides as nanomaterials; using membrane-based nanofiltration techniques; using nanoadsorbent materials as sorbents, nanoclays, and zeolites; and using semiconductors with nanoscale dimensions for nanophotocatalysis to clean up the environment.

Membrane technology has been acknowledged for successfully removing a variety of impurities from water and wastewater for more than three decades. When a driving force is exerted across a membrane, substances are separated by the thin layer of semipermeable material that comprises the membrane. Depending on their chemical and physical characteristics, membranes act as a barrier, preventing some components from passing through [45,67]. Membrane filtration stands out as the most dependable conventional water treatment method because it is quick, easy to scale up, highly effective, and flexible enough to work with other cutting-edge treatment methods. Based on the molecular weight cut-off values (MWCO) of the specific membrane and trans-membrane pressure, the pressure-driven filtering technology is divided into low-pressure MF and UF and high-pressure NF and RO. The ability to remove different sizes of particles from a mixture is the key to understanding these four membrane filtering techniques. Nanofiltration stands out among them as the most environmentally friendly option for removing various pollutants. It is also utilized to soften hard water by eliminating multivalent and divalent ions. The NF membrane has pores with an average size of 0.001 µm, or between 1 and 100 nm. Using NF membranes, various pesticides might be removed from the environment with up to 99.9 % efficiency. NF was used to remove pesticides because it removes molecules with low molecular weights (as low as 100–200 Da). As of today, NF is the only filtration technology known to remove organic pollutants and pesticides [82,84].

The limitations of existing technologies include their high cost, the creation of potentially dangerous toxic compounds, the requirement for high temperatures and pressures, etc. Due to the drawbacks of conventional methods, researchers focus on biological systems and favor green synthesis. Green synthesis is a cost-effective and ecologically friendly process for producing nanostructural materials with configurable topologies, morphologies, and particle size distributions. This field of research is rapidly expanding. Plants are utilized to create nanoparticles for methods of synthesis that have a high potential for heavy metal detoxification and accumulation mechanisms [23,62,27]. Leaf, stem, flower, and seeds from a variety of plants can be used to make the extract. Silver ions are transformed into nanoparticles between 50 and 100 nm in size by *Alternanthes dentata* leaf extract. The organic polymers terpenoid and phenolic are highly effective antioxidants and stabilizers for nanomaterial synthesis. When nanoparticles form, phenolate ions can transmit electrons to the metal ions. For instance, the primary phenolic in clove extract, eugenol, is crucial in the bioreduction of AgNO_3_ and HAuCl_4_ to create nanoparticles. In addition to being made up of polyphenolic compounds, flavonoids include anthocyanin, flavonol, flavone, flavanone, isoflavonoid, chalcone, etc. These compounds have the ability to lower the amount of metal ions during the production of nanoparticles. There is evidence that amino acids can convert metal ions into nanoparticles by binding to them. For the creation of silver nanoparticles, it has been shown that amino acids including cysteine, arginine, methionine, and lysine aid in the binding of silver ions. Plant extracts' ability to bioreduce matter through bioreduction depends on several chemical and physical factors, including pH and temperature [53]. It has been discovered that at low pH levels, the rate of metal ion nucleation becomes extremely low, causing agglomeration to occur in metal nanoparticles. Because of this, there may be a chance that large-sized nanoparticles will form due to low pH levels, whereas higher pH levels can aid in forming small-sized nanoparticles. pH is another factor that affects the form of the nanoparticles produced. The predominant activities of the biomolecules found in plant extract are controlled by the pH of the environment, which has a significant impact on how the biomolecules interact with the metal ions during the creation of nanoparticles [53,61].

Temperature plays a role in the nucleation rate of nanoparticles made from plant extract. The sizes, shapes, and rates of nanoparticle creation change throughout the course of synthesis, along with the reaction temperatures. The incubator's temperature influences the metal ion reduction process, resulting in a shift in hue due to surface Plasmon resonance. Silver nanowires develop at room temperature as a result of the linear accumulation of silver nanoparticles, which causes recrystallization. However, when calcined at 400 °C and subsequently heated, the interaction between biomolecules and the surfaces of silver nanoparticles alters, inhibiting nanoparticle coalescence. As a result, several crooked nanorods and spherical silver nanoparticles are seen. Nucleation depends on reaction temperature in that at high temperatures, the action rate increases and more gold ions are employed to create nuclei, preventing the secondary reduction process on those nuclei's surfaces. Thus, round nanoparticles are produced. Usually, secondary nucleation takes place at low temperatures.

## 6 Microbiological nanotechnology for industrial effluent bioremediation

### 6.1 Microorganisms help advance nanotechnology

The sustainability and environmental friendliness of nanotechnology are increased by using microorganisms concurrently with the biofabrication of nanoparticles. Chemically produced nanoparticles may have substantial downsides because of chemicals and self-agglomeration in aqueous solutions. As a result, employing bacterial, fungal, and plant extracts to create nanoparticles in an environmentally acceptable manner could be a feasible solution. As reductive agents for the metal-complex salt, they generate metallic nanoparticles. These nanoparticles gain improved firmness in an aqueous environment via coprecipitation or by covering the exterior face of the nanoparticles with proteinaceous and bioactive components. Iron oxide nanoparticles made by *Aspergillus tubingensis* (STSP 25) were bio-fabricated and used in the rhizosphere of Avicennia official in the Sundarbans of India [44]. Pb (II), Ni (II), Cu (II), and Zn (II) heavy metals, as well as other contaminants, were removed from wastewater by the synthesized nanoparticles with an efficiency of more than 90% and a regeneration capacity of up to five cycles [85]. Through chemical reactions involving endothermic reactions, the metal ions were chemically adsorbed onto the nanoparticle surfaces. In different studies, iron oxide nanoparticles and exopolysaccharides (EPS) from *Chlorella vulgaris* were co-precipitated [86]. The successful alteration of nanoparticles by functional groups of EPS was discovered by Fourier-transform infrared spectroscopy (FTIR) investigation. Additionally, it was found that 91% of PO43 and 85% of NH4+ were removed by the nanocomposites [87]. *Escherichia* sp. SINT7, a copper resistant strain of bacteria, was used to create copper nanoparticles [88]. The biogenic nanoparticles have been demonstrated to decompose azo dye and textile wastewater. In treated samples, there was a reduction in suspended particles, chloride, and phosphate ions, as well as other components of industrial effluent. The effectiveness of such biogenic nanoparticles increases industries' ability to produce things economically and sustainably. When it comes to generating nanoparticles, *Pseudoalteromonas* sp. CF10–13 provides an environmentally friendly biodegradation method [89]. The production of metal complexes and inflammatory gasses was prevented by endogenous nanoparticle synthesis. It is preferable to employ biogenic particles for industrial effluent cleaning. Apart from directly producing nanoparticles, microbes can boost nanotechnology in a variety of different ways. For example, when paired with nanoparticles, microorganisms may produce catalytic enzymes that aid in wastewater treatment [90].

### 6.2 Magnetic nanoparticles and their immobilizing matrix

Enzymes and nanotechnology work well together to reduce the environmental impact of nanomaterials. Enzyme molecules reduce the contact between their cells and nanomaterials by creating steric hindrances and lowering the surface energy [9]. Enzymes are environmentally beneficial and offer an additional characteristic of catalysis, which increases the adaptability and efficiency of nanomaterials in bioremediation and the creation of renewable energy. In contrast, mounted enzymes on nanomaterials are incredibly stable because they are resistant to unfolding, less susceptible to diffusional restrictions, able to be utilized again, and have improved kinetic properties. Through increased enzyme loading, nanoparticles' wide surface areas enhance immobilization effectiveness. The immobilizing matrix of magnetic nanoparticles, typically utilized, makes extracting immobilized enzymes from the reaction mixture simple. Immobilizing multimeric enzymes, including oxidoreductases, on nanomaterials also helps to stabilize them. Research has demonstrated how well both of these technologies work together [88,89].

The impact of immobilized peroxidase enzymes on wastewater bioremediation was demonstrated by Darwesh et al. (2019). They discovered that iron oxide magnetic nanoparticles modified with glutaraldehyde obtained pH- and temperature-stable immobilised enzymes. Each of the azo dyes, green and red, could be eliminated by the immobilized peroxidase enzyme in 4 h. When both dyes were employed simultaneously in lab-scale studies, it took 6 h to eliminate the dyes completely. Industrial effluents are frequently treated using laccase. Laccase has been immobilized for biodegradation using various composites of magnetic nanoparticles. In a study, as a magnetic carrier for laccase immobilization, a composite made of Fe_3_O_4_ and chitosan was employed. Completing 10 cycles, the covalently linked laccase remained stable and was still able to extract 2, 4-Dichloro- and 4,‑chloro-phenol from the solution. After 12 h, 75.5 % and 91.4 % of 4-CP and 2,4-DCP were broken down, respectively. Accordingly, it is clear from such studies that nanotechnology and enzyme technology offer a stable and effective environment for the breakdown of industrial effluents.

### 6.3 Role of nanoparticles in biohydrogen production from wastewater

New opportunities for producing biohydrogen from wastewater have been made possible by the addition of nanoparticles to fermentative bacteria [91]. To produce biohydrogen, the scientist used a mixed culture of bacteria coupled with a single, dual, and multiple nanoparticle system. They discovered that using numerous nanoparticles increased biohydrogen production by up to 14 % compared to using a single nanoparticle. A greater amount of biohydrogen was produced due to the various nanoparticles' improved hydrogenase and dehydrogenase activity. Similarly, adding nickel oxide and hematite nanoparticles resulted in 1.2–4.5 times more biohydrogen production than using only one kind. Combining nanoparticles led to a maximum hydrogen output of 8.83 mmol/g COD. The enhanced activity of the enzymes hydrogenase and ferredoxin oxidoreductase is responsible for this rise. As a result, nanotechnology can also be employed to provide green energy for sustainable industrial development and environmentally friendly manufacturing.

## 7 Applications of green nanoparticles in treating industrial wastewater

There are numerous uses for green nanotechnology in various industries. To cleanse many types of wastewaters, yet, green produced metal or metal oxide nanoparticles have been widely used [92]. Silver nanoparticles were created using *P. thonningii* leaf extract in a research effort, and they were utilized successfully to remove heavy metals from simulated wastewater [93]. Researchers have reported a green method for making silver nanocomposites for treating textile dyes using *Ocimum tenuiflorum* leaf extract [94]. Wastewater that has been treated can be effectively used in both home and commercial settings. According to another study, tangerine peel extract created green synthetic iron oxide nanoparticles that effectively removed cadmium from wastewater [95]. Tangerine peel extract was employed in the current research as a stabilizing agent, which contributed to the low-cost and environmentally friendly synthesis of iron oxide nanoparticles. This is because it is crucial for controlling the size and morphology of nanoparticles during synthesis. By revealing the presence of photochemicals in leaf extract, nanoparticles may be stabilized or capped. In addition, the first application of green-synthesised TiO2 NPs demonstrated their capacity to simultaneously remove chromium (Cr) and chemical oxygen demand (COD) from secondary-treated TWW. This work removed 82.26 % of COD and 76.48 % of Cr from tannery wastewater using green-synthesized TiO2 NPs in a self-designed and manufactured parabolic trough reactor (PTR). Employing *Lagerstroemia speciosa* leaf extract, a quick green chemistry process was used to create AuNPs [96]. When dyes including methylene blue, methyl orange, bromophenol blue, bromocresol green, and 4-nitrophenol were reduced under visible light in the presence of NaBH4, the green method for the manufacture of AuNPs demonstrated photocatalytic solid activity. An active and exciting field of research is the creation of functional supramolecular structures using renewable and natural materials. As was noted before, few studies have been done that consider extracting nanostructures and NPs from plants, despite the fact that many studies have been done that are connected with the synthesis and isolation of metallic NPs. These nanomaterials can create biocompatible and bioactive nanodevices for brand-new prospective applications [97]. Plants can serve as sustainable, biorenewable, diverse supplies and platforms for the creation of valuable nanoparticles and nanostructures that have both nontoxic and biocompatible qualities [98]. Increased population expansion, commercialization, and industrialization all contribute to the deterioration of water quality.

## 8 Green nanotechnology's drawbacks

Green nanotechnology has its own set of difficulties and is still in its infancy. The primary obstacles that green nanotechnology must overcome are as follows: (i) dealing with toxicity concerns related to nanomaterials; (ii) technical and financial obstacles; (iii) regulations controlling processes employed in nanomanufacturing; (iv) implementing scaling-up techniques; and (v) life cycle analyses [99,[100], [101], [102], [103]]. For green and sustainable growth, the aforementioned recommendations need to be carefully examined. The main drawbacks of this technology are the expenses and hazards involved in creating low-pollution, environmentally safe products based on nanotechnology. The costs and risks associated with producing ecologically safe, low-pollution nanotechnology-based goods are the key downsides of this technology. Although green nanotechnology has come a long way, concerns about the long-term viability of its more environmentally friendly uses persist. Green nanotechnology-based products can be efficiently produced, but the upstream processing of those products is where the most significant safety concerns reside. Greener nanoproducts are currently the subject of research for their creation and use, although only a few have hit the market [99,104,105]. The common consensus is that it will take some time before the potential of green nanotechnology is completely understood.

## 9 Conclusion

Nanoparticles and green chemistry can be considered an environmentally responsible approach in treating wastewater and escalating the hardships in managing contaminants. The rapid removal of organic and inorganic pollutants, such as heavy metal ions and dyes, from wastewater is made possible on a large scale due to the widespread application of green nanotechnology. In the upcoming times, nanomaterials are expected to upgrade the current treatment procedures by increasing their effectiveness and reusability which inturn can reduce the cost of maintaining industrial applications. We could see that nanoparticles exhibit unique properties that can be harnessed for treating contaminated water. Many current remediation procedures involved are energy-intensive and unprofitable because they aren't capable of completely cleaning the wastewater. Using green synthesized NPs to treat wastewater is not only a sustainable choice but also a promising technology for developing nations that may achieve the goal of zero effluent discharge following wastewater treatment utilizing lesser energy resources. As discussed we are already advancing with our efficient techniques to synthesize nanoparticles. Their significant anti-fungal, anti-bacterial and water remediation nature is being utilised. This alongwith green chemistry has made the approach much more safer and efficient. Green nanotechnology presents a fantastic prospect for aiding in the resolution of advancing sustainable development. In the future, the emerging field of nanotechnology will be required to be developed sustainably, and whole-system thinking must be applied to assess the environmental implications of nanoproducts. In order to determine their contribution to the green growth, it is important to properly consider all issues like potential life cycle assessment of freshly synthesized nanoproducts before they are released to the commercial market. Although there is always room for improvement, using the concepts of green chemistry to identify better products and processes is promising. The synergy between nanoparticles and green chemistry offers path for a cleaner and more sustainable future of our planet. Moving forward, it is imperative to continue research in fostering international collaborations and developing new technologies to ensure safe, clean and drinkable water resources for our upcoming generations by the use of nanotechnology.

## CRediT authorship contribution statement

**Shreya Rathod:** Data curation. **Subham Preetam:** Conceptualization, Funding acquisition, Investigation, Methodology, Project administration, Resources, Software, Supervision, Validation, Visualization, Writing – original draft, Writing – review & editing. **Chetan Pandey:** Investigation, Methodology, Project administration, Resources. **Sweta Parimita Bera:** Conceptualization, Data curation, Formal analysis, Investigation.

## Declaration of competing interest

The authors declare no competing financial interests.

## Data Availability

No data was used for the research described in the article.

## References

[1] Bucs S.S., Farhat N., Fortunato L. (2022). Water treatment process. Membranes (Basel).

[2] He C. (2021). Future global urban water scarcity and potential solutions. Nat. Commun..

[3] Rajendran S., Wanale S.G., Gacem A., Yadav V.K., Ahmed I.A., Algethami J.S., Kakodiya S.D., Modi T., Alsuhaibani A.M., Yadav K.K., Cavalu S. (2023). Nanostructured iron oxides: structural, optical, magnetic, and adsorption characteristics for cleaning industrial effluents. Crystals (Basel).

[4] Saeed M.U. (2022). Microbial bioremediation strategies with wastewater treatment potentialities - a review. Sci. Total. Environ..

[5] Maryam S., Ul Haq I., Yahya G., Ul Haq M., Algammal A.M., Saber S., Cavalu S. (2023). COVID-19 surveillance in wastewater: an epidemiological tool for the monitoring of SARS-CoV-2. Front. Cell Infect. Microbiol..

[6] Bhat S.A. (2022). Sustainable nanotechnology based wastewater treatment strategies: achievements, challenges and future perspectives. Chemosphere.

[7] Manikandan S. (2021). Emerging nano-structured innovative materials as adsorbents in wastewater treatment. Bioresour. Technol..

[8] Choudhary N., Yadav V.K., Ali H., Ali D., Almutairi B.O., Cavalu S., Patel A. (2023). Remediation of methylene blue dye from wastewater by using zinc oxide nanoparticles loaded on nanoclay. Water (Basel).

[9] Arenas-Sanchez A., Rico A., Vighi M. (2016). Effects of water scarcity and chemical pollution in aquatic ecosystems: state of the art. Sci. Total. Environ..

[10] Bishoge O.K. (2018). Remediation of water and wastewater by using engineered nanomaterials: a review. J. Environ. Sci. Health a Tox. Hazard. Subst. Environ. Eng..

[11] Saratale R.G. (2018). Exploiting fruit byproducts for eco-friendly nanosynthesis: citrus x clementina peel extract mediated fabrication of silver nanoparticles with high efficacy against microbial pathogens and rat glial tumor C6 cells. Environ. Sci. Pollut. Res. Int..

[12] Saratale R.G. (2018). A comprehensive review on green nanomaterials using biological systems: recent perception and their future applications. Colloids. Surf. B Biointerfaces..

[13] Poornima S. (2022). Emerging nanotechnology based advanced techniques for wastewater treatment. Chemosphere.

[14] Wiesner M. (2013). Progress towards the responsible application of nanotechnology for water treatment. Water. Res..

[15] Qu X., Alvarez P.J., Li Q. (2013). Applications of nanotechnology in water and wastewater treatment. Water. Res..

[16] Ajith M.P. (2021). Recent innovations of nanotechnology in water treatment: a comprehensive review. Bioresour. Technol..

[17] Zahmatkesh S. (2022). A comprehensive review of various approaches for treatment of tertiary wastewater with emerging contaminants: what do we know?. Environ. Monit. Assess..

[18] Bora T., Dutta J. (2014). Applications of nanotechnology in wastewater treatment–a review. J. Nanosci. Nanotechnol..

[19] Sun M., Gupta A.S (2020). Vascular nanomedicine: current status, opportunities, and challenges. Semin. Thromb. Hemost..

[20] Thangavelu L., Veeraragavan G.R. (2022). A survey on nanotechnology-based bioremediation of wastewater. Bioinorg. Chem. Appl..

[21] Kim M. (2019). Green-synthesis of anisotropic peptone-silver nanoparticles and its potential application as anti-bacterial agent. Polymers (Basel).

[22] Kumar S. (2014). Nanotechnology-based water treatment strategies. J. Nanosci. Nanotechnol..

[23] Jain K. (2021). Nanotechnology in wastewater management: a new paradigm towards wastewater treatment. Molecules..

[24] Jiang M. (2018). The role of nanomaterials and nanotechnologies in wastewater treatment: a bibliometric analysis. Nanoscale Res. Lett..

[25] Ahmed S.N., Haider W. (2018). Heterogeneous photocatalysis and its potential applications in water and wastewater treatment: a review. Nanotechnology.

[26] Moreira N.F.F. (2018). Solar treatment (H(2)O(2), TiO(2)-P25 and GO-TiO(2) photocatalysis, photo-Fenton) of organic micropollutants, human pathogen indicators, antibiotic resistant bacteria and related genes in urban wastewater. Water. Res..

[27] Kajitvichyanukul P. (2022). Challenges and effectiveness of nanotechnology-based photocatalysis for pesticides-contaminated water: a review. Environ. Res..

[28] Ahmed S.N., Haider W. (2018). Heterogeneous photocatalysis and its potential applications in water and wastewater treatment: a review. Nanotechnology.

[29] Kanakaraju D., Glass B.D., Oelgemöller M. (2014). Titanium dioxide photocatalysis for pharmaceutical wastewater treatment. Environ. Chem. Lett..

[30] Sinar Mashuri S.I., Ibrahim M.L., Kasim M.F., Mastuli M.S., Rashid U., Abdullah A.H., Islam A., Asikin Mijan N., Tan Y.H., Mansir N. (2020). Mohd Kaus NH. Photocatalysis for organic wastewater treatment: from the basis to current challenges for society. Catalysts..

[31] Mali S.C., Dhaka A., Githala C.K., Trivedi R. (2020). Green synthesis of copper nanoparticles using Celastrus paniculatus Willd. leaf extract and their photocatalytic and antifungal properties. Biotechnol. Reports.

[32] Religa P., Kowalik A., Gierycz P. (2011). Application of nanofiltration for chromium concentration in the tannery wastewater. J. Hazard. Mater..

[33] Theodorakopoulos G.V. (2023). Novel pilot-scale photocatalytic nanofiltration reactor for agricultural wastewater treatment. Membranes (Basel).

[34] Arumugham T. (2021). Recent developments in porous ceramic membranes for wastewater treatment and desalination: a review. J. Environ. Manage..

[35] Gholami F. (2022). Color removal from wastewater using a synthetic high-performance antifouling GO-CPTMS@Pd-TKHPP/polyether sulfone nanofiltration membrane. Environ. Sci. Pollut. Res. Int..

[36] Cabrera S.M. (2022). Performance evaluation of an industrial ceramic nanofiltration unit for wastewater treatment in oil production. Water. Res..

[37] Vunain E., Mishra A.K., Mamba B.B. (2016). Dendrimers, mesoporous silicas and chitosan-based nanosorbents for the removal of heavy-metal ions: a review. Int. J. Biol. Macromol..

[38] Gautam R.K., Tiwari I. (2020). Humic acid functionalized magnetic nanomaterials for remediation of dye wastewater under ultrasonication: application in real water samples, recycling and reuse of nanosorbents. Chemosphere.

[39] Ahmed S.F. (2022). Nanomaterials as a sustainable choice for treating wastewater. Environ. Res..

[40] Xu P. (2012). Use of iron oxide nanomaterials in wastewater treatment: a review. Sci. Total. Environ..

[41] Paiva-Santos A.C. (2021). Plant-mediated green synthesis of metal-based nanoparticles for dermopharmaceutical and cosmetic applications. Int. J. Pharm..

[42] Jiang Z. (2022). Progress in laser ablation and biological synthesis processes: "top-down" and "bottom-up" approaches for the green synthesis of au/ag nanoparticles. Int. J. Mol. Sci..

[43] Carnide G. (2023). Secured nanosynthesis-deposition aerosol process for composite thin films incorporating highly dispersed nanoparticles. Adv. Sci. (Weinh).

[44] Low S.S. (2022). Sonoproduction of nanobiomaterials - a critical review. Ultrason. Sonochem..

[45] Asmat-Campos D., de Oca-Vásquez G.M., Rojas-Jaimes J., Delfín-Narciso D., Juárez-Cortijo L., Nazario-Naveda R., de la Cruz M.S. (2023). Cu2O nanoparticles synthesized by green and chemical routes, and evaluation of their antibacterial and antifungal effect on functionalized textiles. Biotechnol. Reports.

[46] Pescuma M., Aparicio F., Zysler R.D., Lima E., Zapata C., Marfetán J.A., Ordoñez O.F. (2023). Biogenic selenium nanoparticles with antifungal activity against the wood-rotting fungus Oligoporus pelliculosus. Biotechnol. Reports.

[47] Din M.I. (2017). Green adeptness in the synthesis and stabilization of copper nanoparticles: catalytic, antibacterial, cytotoxicity, and antioxidant activities. Nanoscale Res. Lett..

[48] Karvekar O.S. (2022). Biogenic synthesis of silver anchored ZnO nanorods as nano catalyst for organic transformation reactions and dye degradation. Appl. Nanosci..

[49] Wang J. (2020). Enhanced thermoelectric performance in n-Type SrTiO(3)/SiGe composite. ACS. Appl. Mater. Interfaces..

[50] Cho W. (2023). Synthesis of colloidal gan and aln nanocrystals in biphasic molten salt/organic solvent mixtures under high-pressure ammonia. ACS Nano.

[51] Penza M. (2010). Metal-modified and vertically aligned carbon nanotube sensors array for landfill gas monitoring applications. Nanotechnology.

[52] Liu H. (2016). Morphology controlling method for amorphous silica nanoparticles and jellyfish-like nanowires and their luminescence properties. Sci. Rep..

[53] Chen Y. (2021). Enhancing robustness of activated sludge with Aspergillus tubingensis as a protective backbone structure under high-salinity stress. J. Environ. Manage.

[54] Nguyen M.T., Deng L., Yonezawa T. (2021). Control of nanoparticles synthesized via vacuum sputter deposition onto liquids: a review. Soft. Matter..

[55] Sergievskaya A., Alem H., Konstantinidis S. (2023). Magnetron sputtering onto nonionic surfactant for 1-step preparation of metal nanoparticles without additional chemical reagents. Nanotechnology.

[56] Bodin A. (2018). Engineering Ni-Mo-S Nanoparticles for Hydrodesulfurization. Nano Lett..

[57] Mahadevaiah (2019). Laser-ablation-synthesized nanoparticles and animal cell lines studies. J. Biosci..

[58] Perez-Tanoira R. (2022). Silver nanoparticles produced by laser ablation and re-irradiation are effective preventing peri-implantitis multispecies biofilm formation. Int. J. Mol. Sci..

[59] Blazeka D., Car J., Krstulovic N. (2022). Concentration Quantification of TiO(2) nanoparticles synthesized by laser ablation of a Ti target in water. Materials (Basel).

[60] Subhan A., Mourad A.I., Al-Douri Y. (2022). Influence of laser process parameters, liquid medium, and external field on the synthesis of colloidal metal nanoparticles using pulsed laser ablation in liquid: a review. Nanomaterials (Basel).

[61] Zhang J. (2017). Colloidal metal nanoparticles prepared by laser ablation and their applications. Chemphyschem..

[62] Lee S.J. (2022). Nanogap-tailored Au nanoparticles fabricated by pulsed laser ablation for surface-enhanced Raman scattering. Biosens. Bioelectron..

[63] Popov A.A. (2022). Synthesis of titanium nitride nanoparticles by pulsed laser ablation in different aqueous and organic solutions. Nanomaterials (Basel).

[64] Panneer N.K. (2023). Ecofriendly sol-gel-derived dye-sensitized solar cells with aluminium-doped tin oxide photoanode. Environ. Sci. Pollut. Res. Int..

[65] Krupa A.N., Vimala R. (2016). Evaluation of tetraethoxysilane (TEOS) sol-gel coatings, modified with green synthesized zinc oxide nanoparticles for combating microfouling. Mater. Sci. Eng. C. Mater. Biol. Appl..

[66] Bhullar S., Goyal N., Gupta S. (2022). Synthesizing and optimizing rutile TiO(2) nanoparticles for magnetically guided drug delivery. Int. J. Nanomed..

[67] Batool T. (2020). Microwave assisted sol-gel synthesis of bioactive zirconia nanoparticles - correlation of strength and structure. J. Mech. Behav. Biomed. Mater..

[68] Sebti Y. (2022). Assessment of the morphological, optical, and photoluminescence properties of HfO(2) nanoparticles synthesized by a sol-gel method assisted by microwave irradiation. Inorg. Chem..

[69] Majidi S. (2016). Current methods for synthesis of magnetic nanoparticles. Artif. Cells Nanomed. Biotechnol..

[70] Birch D.J., Yip P. (2014). Nanometrology. Methods Mol. Biol..

[71] Thomsen T. (2021). Covalent and noncovalent conjugation of degradable polymer nanoparticles to T lymphocytes. Biomacromolecules.

[72] Majeric P., Rudolf R. (2020). Advances in ultrasonic spray pyrolysis processing of noble metal nanoparticles-review. Materials (Basel).

[73] Ahmad S. (2019). Green nanotechnology: a review on green synthesis of silver nanoparticles - an ecofriendly approach. Int. J. Nanomed..

[74] Chaudhary R.G., Singh N.B. (2023). Green nanomaterials: a road map to safe nanotechnology. Curr. Pharm. Biotechnol..

[75] Yashveer S. (2014). Green biotechnology, nanotechnology and bio-fortification: perspectives on novel environment-friendly crop improvement strategies. Biotechnol. Genet. Eng. Rev..

[76] Salem S.S. (2023). A mini review on green nanotechnology and its development in biological effects. Arch. Microbiol..

[77] Parthiban E., Manivannan N., Ramanibai R., Mathivanan N. (2019). Green synthesis of silver-nanoparticles from Annona reticulata leaves aqueous extract and its mosquito larvicidal and anti-microbial activity on human pathogens. Biotechnol. Reports.

[78] Ali N.H., Mohammed A.M. (2021). Biosynthesis and characterization of platinum nanoparticles using Iraqi Zahidi dates and evaluation of their biological applications. Biotechnol. Reports.

[79] Kshtriya V., Koshti B., Gour N., Verma S.K., Das A.K. (2021). Comprehensive Analytical Chemistry.

[80] Garcia-Quintero A., Palencia M. (2021). A critical analysis of environmental sustainability metrics applied to green synthesis of nanomaterials and the assessment of environmental risks associated with the nanotechnology. Sci. Total. Environ..

[81] Bera S.P., Tank S.K. (2021). Bioremedial approach of Pseudomonas stutzeri SPM-1 for textile azo dye degradation. Arch. Microbiol..

[82] Yadav V.K. (2022). Recent advances in synthesis and degradation of lignin and lignin nanoparticles and their emerging applications in nanotechnology. Materials. (Basel).

[83] Bera S.P., Godhaniya M., Kothari C. (2022). Emerging and advanced membrane technology for wastewater treatment: a review. J. Basic Microbiol..

[84] Saravanan A. (2022). A review on synthesis methods and recent applications of nanomaterial in wastewater treatment: challenges and future perspectives. Chemosphere.

[85] Taghizadeh S.M. (2022). A study ofl-lysine-stabilized iron oxide nanoparticles (IONPs) on microalgae biofilm formation of Chlorella vulgaris. Mol. Biotechnol..

[86] Grasso G., Zane D., Dragone R. (2019). Microbial nanotechnology: challenges and prospects for green biocatalytic synthesis of nanoscale materials for sensoristic and biomedical applications. Nanomaterials (Basel).

[87] Noman M. (2020). Use of biogenic copper nanoparticles synthesized from a native Escherichia sp. as photocatalysts for azo dye degradation and treatment of textile effluents. Environ. Pollut..

[88] Cheng S. (2019). Biodegradation of metal complex Naphthol Green B and formation of iron-sulfur nanoparticles by marine bacterium Pseudoalteromonas sp CF10-13. Bioresour. Technol..

[89] Jeevanandam J. (2022). Green approaches for the synthesis of metal and metal oxide nanoparticles using microbial and plant extracts. Nanoscale.

[90] Malik S. (2022). Exploring microbial-based green nanobiotechnology for wastewater remediation: a sustainable strategy. Nanomaterials (Basel).

[91] Hano C., Abbasi B.H. (2021). Plant-based green synthesis of nanoparticles: production, characterization and applications. Biomolecules.

[92] Kamaraj C. (2023). Green synthesis of silver nanoparticles from Cassia Auriculata: targeting antibacterial, antioxidant activity, and evaluation of their possible effects on saltwater microcrustacean, Artemia Nauplii (non-target organism). Sci. Total. Environ..

[93] Tailor G. (2020). Green synthesis of silver nanoparticles using Ocimum canum and their anti-bacterial activity. Biochem. Biophys. Rep..

[94] Ehrampoush M.H. (2015). Cadmium removal from aqueous solution by green synthesis iron oxide nanoparticles with tangerine peel extract. J. Environ. Health Sci. Eng..

[95] Choudhary B.C. (2017). Photocatalytic reduction of organic pollutant under visible light by green route synthesized gold nanoparticles. J. Environ. Sci. (China).

[96] Benelli G. (2017). Nanoparticles as effective acaricides against ticks-a review. Ticks. Tick. Borne Dis..

[97] Sahoo A. (2022). Microbased biorefinery for gold nanoparticle production: recent advancements, applications and future aspects. Prep. Biochem. Biotechnol..

[98] Benelli G. (2016). Green synthesized nanoparticles in the fight against mosquito-borne diseases and cancer-a brief review. Enzyme Microb. Technol..

[99] Nahak B.K. (2022). Advances in organ-on-a-chip materials and devices. ACS. Appl. Bio Mater..

[100] Bhattacharjee R. (2022). The emergence of metal oxide nanoparticles (NPs) as a phytomedicine: a two-facet role in plant growth, nano-toxicity and anti-phyto-microbial activity. Biomed. Pharmacother..

[101] Bhattacharjee R. (2023). Nanotheranostics to target antibiotic-resistant bacteria: strategies and applications. OpenNano.

[102] Malik S. (2022). Exploring microbial-based green nanobiotechnology for wastewater remediation: a sustainable strategy. Nanomaterials.

[103] Preetam S. (2022). Bio-Nano Interface: Applications in Food, Healthcare and Sustainability.

[104] Kumari P. (2023). Advanced and Innovative Approaches of Environmental Biotechnology in Industrial Wastewater Treatment.

[105] Sikiru S., Abiodun O.A., Sanusi Y.K., Sikiru Y.A., Soleimani H., Yekeen N., Haslija A.A. (2022). A comprehensive review on nanotechnology application in wastewater treatment a case study of metal-based using green synthesis. J. Environ. Chem. Eng..

